# Interdependent Determinants of Health and Death? Examining the Linkages between Health Equity, Human Rights, and Democracy during COVID-19

**DOI:** 10.5334/aogh.4104

**Published:** 2023-09-18

**Authors:** Lisa Forman, Carly Jackson

**Affiliations:** 1 Dalla Lana School of Public Health, The University of Toronto, 155 College Street, Toronto, ON, Canada M5T 3M7, CA

**Keywords:** COVID-19, democracy, human rights, equity, global health

## Abstract

**Background:**

The COVID-19 pandemic has been characterised by health inequities in differential rates of COVID-19-related morbidity and mortality and differential access to essential COVID-19-related health care interventions such as vaccines. Inequities through the pandemic have deeply illuminated the interdependence between health inequities, human rights, and democratic leadership and the imperative to delve more deeply into these key determinants of health, illness, and death.

**Methods:**

In this paper, we consider what COVID-19 suggests we should be learning about the relationships between democracy, human rights, and health equity. We first elaborate on the growing prominence of the framework and discourse of health equity. We turn to elaborate on a longer-standing trend of democratic backsliding and populist leadership during COVID-19. We consider human rights violations and domestic and global inequities that have characterised COVID-19 and COVID responses.

**Findings and conclusions:**

The pandemic has illustrated how rights-violating, negligent, and inequitable political leadership can deeply determine health outcomes. It has equally shown how democratic norms and institutions, including human rights and equity, offer discourse, standards, and tools that can be effectively used to challenge inequitable leadership on health. More fundamentally, it underscores how great the need is for approaches to public health emergencies rooted in human rights, equity, and good governance, including through a pandemic treaty in negotiation.

## Introduction

The COVID-19 pandemic is forcing a reckoning with the relationship between democracy, human rights, and health equity. Data on global COVID-19 infections, hospitalisations, and deaths reveal distinct inequities within and between countries when it comes to differential rates of COVID-19-related morbidity and mortality and differential access to essential COVID-19-related health care interventions, such as vaccines. The primary paradigm in global health scholarship for exploring these disparities is through the discourse of ‘health equity,’ a conceptual framing that has come to dominate global health scholarship and practice in the past two decades, and which incorporates and expands upon the social determinants of health. To a lesser extent, COVID-19-related disparities are explored within a human rights framework or within the context of democratic backsliding (or ‘autocratisation’). Very rarely are the connections between democracy (and especially democratic leadership), human rights, and health equity explored in general or in relation to COVID-19. Yet, COVID-19 has deeply illuminated the interdependence between health inequities, the protection and abuse of human rights, and political leadership and has underscored the imperative to delve more deeply into these key determinants of health, illness, and death [1].

Accordingly, we consider what this global pandemic suggests we should be learning about the relationships between democracy, human rights, and health equity. We first elaborate on the growing prominence of the framework and discourse of health equity. We turn to elaborate on a longer-standing trend of democratic backsliding and populist leadership during COVID-19. We then turn to consider human rights violations and domestic and global inequities that have characterised COVID-19 and COVID responses. We close with thoughts about what broader connections between these domains might imply for research, policy, and governance of future global health emergencies.

### The emergence of the paradigm of health equity

Before the early 2000s, global health research (then called ‘international health’) was characterised in theory and practice as Northern researchers researching and responding to health challenges in the Global South [2,3]. ‘International health’ research tended to adopt a narrow focus on technical fixes through vertical programmes and a predominant focus on infectious diseases to the exclusion of broader determinants of health inequity [3]. By the early to mid-2000s, the language of ‘international’ health research began to give way to that of ‘global’ health research, reflecting the contemporary focus on globalisation and its health impacts [4,5]. Globalisation’s parallel growth in interdependence and inequality was viewed as creating a ‘logic of health interdependence’, requiring global solidarity to redress inequities and realise human rights [6].

Yet, it was not until after the seminal 2008 report of the Commission on the Social Determinants of Health (CSDH) that the language and conceptualisation of ‘health equity’ came to dominate the discourse and practice of global health research. Certainly, the concept of health equity had deeper historical roots in the creation of the World Health Organization (WHO) in the 1940s, in the Declaration of Alma Ata in 1978, in the New International Economic Order emerging in the 1970–1980s, and in the AIDS treatment campaigns that came to prominence in the early to mid-2000s [7,8,9]. The 2008 CSDH report drew from and built on this history to reframe health equity beyond individual human behaviour and discrete health issues within the health system towards the far bigger picture of the social determinants of health. The CSDH described the social determinants of health as the conditions in which people work, live, and play, the social systems in place to address health and illness, and the political, social, and economic systems that shape those conditions and systems [10]. The report reframed the imperative to address these health inequities as an ethical imperative rooted not in philanthropy but in social justice and human rights [10].

The discursive impact of the 2008 CSDH is evident in contemporary public and global health policy and scholarship. In sharp contrast to the Millennium Development Goals (MDGs) that came before them, the 2015 Sustainable Development Goals (SDGs) employ a wider health equity lens, with a free-standing goal of reducing inequality within and among countries (SDG 10) and a system-wide goal of achieving universal health coverage globally (SDG 3.8), a health goal implicitly rooted in equitable access to health care. This rhetoric has equally taken root in domestic health policy: a 2020 report on the state of public health in Canada took an equity approach to the COVID-19 pandemic explicitly rooted in the conceptual framework advanced in the CSDH report and focused on injustices like stigma and discrimination as key drivers of health inequity broadly and COVID-19 specifically [11].

**Figure 1:**
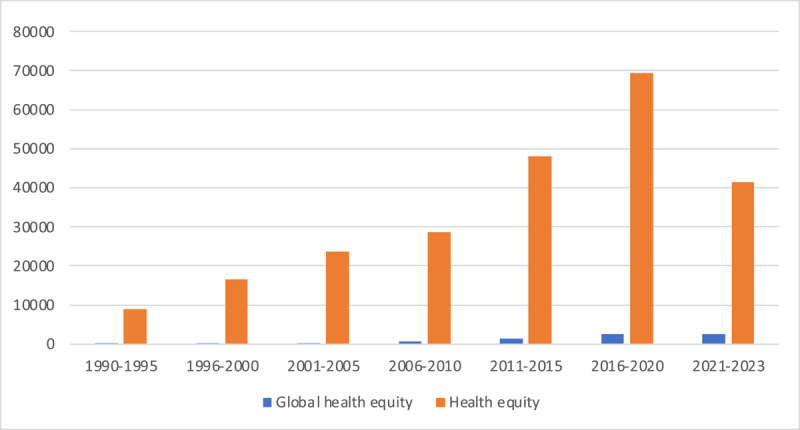
The growth of ‘health equity’ scholarship. **Source:** Google scholar search 2023.

The growing prominence of the discourse of health equity is especially apparent in scholarly research [12]. As the Figure 1 below indicates, since 1990, there have been increases in the use of the term ‘health equity’ in scholarship. Unsurprisingly, these increases have been exponential since 2020 and the start of the pandemic. At the same time, and especially since COVID-19, there has also been a tremendous expansion in literature exploring the social, political, legal, and commercial determinants of health [13,14,15,16,17]. These trends underscore a growing awareness of the risks of global interdependence and of the need for language, principles, and tools to measure and respond to the less positive health impacts of growing global interdependence.

### Autocratisation, populism, and health

It is perhaps not surprising that growing awareness of the negative impacts of global interdependence has coincided with a populist backlash against ‘globalism’ and a retreat from democracy. For over sixteen years, the world has been seeing a consistent and intensifying democratic retreat [18,19], marking the first time since the Second World War that more countries are autocratising than democratising [20]. In 2022 alone, sixty countries suffered democratic declines, with only 20 percent of the global population now understood to live in free countries [19]. Moves towards autocracy may be gradual, such that some nations are considered ‘electoral autocracies’, where ‘elections are held only under conditions that prevent opposition parties from fairly campaigning and votes are prevented from being freely cast or accurately counted’ [20].

The health impacts of democratic retreat were apparent even before COVID-19, with one study finding that autocratising countries had made less progress on expanding health services under universal health coverage and that their citizens faced higher out-of-pocket spending, at least in part because ‘democratic erosion modifies the willingness and capacity for governments to respond to the health needs of citizens’ [20]. Another study found that the ‘welfare chauvinistic ideology’ of populist parties (where welfare benefits are provided to a hypothetical ‘in-group’ while being restricted for the ‘out-group’) is harmful for public health [21].

With the COVID-19 pandemic, the impacts of populist governance on COVID-19 and health have become even more apparent. Over significant periods of the pandemic, the WHO’s COVID-19 dashboard, which tracks daily COVID-19 rates of infection and death across the globe since the latter half of 2020, reported the highest rates of infection and death in the United States, India, Brazil, and Russia [22]. McKee et al. suggest that these figures suggest ‘a striking correlation between countries led by politicians who support populist messages and the poor performance in responding to COVID-19’, with the United States, Brazil, Russia, India, and the United Kingdom in 2020 accounting for 51% of all cases worldwide but only 27% of the world’s population [23]. In Manaus, Brazil, by March 2021, over 10 percent of all people over 85 years of age had died of COVID-19. In the US, COVID-19 became the leading cause of death for several months in late 2020 and early 2021 [24], and it has been estimated that up to 40% of COVID-19 fatalities that occurred by February 2021 in the US could have been prevented [25]. These outcomes are in stark contrast with countries that have had much more successful COVID-19 responses, such as Taiwan, South Korea, New Zealand, and Australia, where little or no excess mortality was recorded [24]. Jennifer Prah Ruger and Amartya Sen suggest that countries like these shared a willingness to govern ‘for the common good, shared responsibility for scientifically grounded systems, rational, compassionate and transparent communication and ethical leadership and trust’ [26]. They argue that COVID-19 underscores that the ‘connection between democracy and health is multidirectional and based on social justice’ and on ethical leadership [26].

In contrast, populist governance on COVID-19 has tended to ‘blame “others” for the pandemic, such as immigrants and the Chinese government; deny evidence and show contempt for institutions that generate it; and portray themselves as the voice of the common people against an out-of-touch “elite”’ [23]. In the US, Trump’s presidency showcased a disregard and antipathy for democratic norms, human rights, international law, and global solidarity, which, under COVID-19, coalesced around contestation of the legitimacy of the World Health Organization and undermining of public health practice, evidence, and science, with dire impacts on public health efforts to control the spread of COVID-19 in the US. The US’s outsized political and cultural influence globally ensured that these governance and democracy failures set an insidious norm for both right-wing populist leaders like Jair Bolsonaro in Brazil and Narendra Modi in India, and left-wing populists like Lopez Obrador in Mexico. In Brazil, Bolsonaro sustained a denialist stance on COVID-19, characterised by misinformation about the symptoms, risks, and cures of the virus and active flouting of COVID-19 measures [27]. In India, Modi refused to halt a religious pilgrimage in which millions gathered at the Ganges River throughout April 2021 [28] and blamed Muslims for the spread of the virus [29]. In Mexico, Obrador’s response to COVID-19 was reported to be characterised by, among other things, antipathy towards expert scientific knowledge outside the government [30]. These policy responses are not just antithetical to equity and human rights but also appear to be deeply ineffective from a public and global health perspective.

### Health inequities and human rights violations during COVID

Beyond negligent public health governance like this, health outcomes and governance during COVID-19 have been characterised by inequities and human rights violations in multiple ways. In many places, the risk of COVID-19 infection and more severe illness and death has correlated not simply with an income gradient according to demographic factors typically associated with respiratory illness, such as age and health status, but with factors like race and disability. In Toronto, the largest metropolitan city in Canada’s most populous province, 80% of COVID-19 infections occurred in racialised populations, while these populations make up only 52% of the city’s population [31,32]. In the UK, black people were four times more likely to die from COVID-19 than white people [33] and in the United States, racialised groups were more likely to be infected, hospitalised, and subsequently die from COVID-19 than their white counterparts [34]. Multiple scholars have illustrated how state responses to COVID-19 ‘magnified several structural inequalities and patterns of discrimination across gender, race, age, and sexual orientation’ [35]. These disproportionate impacts have arisen due to the ways in which social and political determinants of health intersect with social bias to increase exposure to the virus, fuelled by the prevalence of underlying medical conditions and decreased access to health care among racialised populations in these regions [11,36]. It is likely not a coincidence that in both Canada and the UK there were controversies over collecting and disclosing data that illustrated these trends [31,37,38,39,40]. Health inequities associated with COVID-19 infection rates among racialised and marginalised communities are similarly evident in the use of force in enforcing COVID-19 measures amongst these communities. Data from New York City highlights that 80% of court summonses for failing to adhere to social distance orders went to people of colour [41]. These trends are also reflected in countries such as India, South Africa, and Kenya, where people have been threatened with violence by authorities and killed when live ammunition has been used for crowd control [42,43].

Given the deep interdependence between just governance and health inequities, the pandemic saw wide-spread violations of human rights perpetrated in the name of COVID-19. A Human Rights Measurement Initiative (HRMI) survey of practitioners within human rights organisations (including non-governmental organisations, lawyers, and journalists) reported negative impacts on a wide range of human rights in 2020, with participants identifying the worst impacts on ‘rights to opinion and expression, assembly and association, food, education, health and work’ [44]. The pandemic offered a pretext for outright human rights abuses, including authoritarian power grabs, crackdowns on civil society, and police violence. Most egregiously, within weeks of COVID-19 being declared a pandemic, Viktor Orban, the Prime Minister of Hungary, enacted an indefinite state of emergency, which, with a brief interruption, has been renewed multiple times since then [45,46]. In Uganda, security forces used COVID-19 as an excuse to break up political gatherings and clamp down on the opposition and the media [47]. In South Africa and Kenya, police used disproportionate force to enforce lockdowns, resulting in multiple fatalities [48,49].

Beyond outright abuses like these, COVID measures frequently exceeded the bounds of legitimacy, with a 2020 global monitor finding that by November 2020, more than 61% of all the countries in the world had implemented COVID-19 measures that were concerning from a democracy and human rights perspective because they were ‘either disproportionate, illegal, indefinite or unnecessary in relation to the health threat’ [50]. At the same time, health systems in high- and low-income countries struggled to provide adequate COVID-19 testing, tracing, and treatment, with non-COVID-19 healthcare-restricted, vulnerable populations at high risk of infection and negative health and social impacts, and lockdowns exacerbating poverty, domestic violence, and mental health problems [51].

Disparities in access to COVID-19 vaccines in low- and middle-income countries have emerged as this pandemic’s singular human rights and equity challenge [52]. In high- and upper-middle-income countries, almost two-thirds of people are fully vaccinated, while in low-income countries, this figure falls to below 20% [53]. In Africa, access in many countries falls well below 10–15% [53]. Global political and institutional failures to remediate vaccine inequity during the pandemic have underscored the imperative for a human rights and equity approach during a pandemic [52]. In the absence of this kind of systemic reform, access disparities are likely to continue to be a feature of global responses in future health emergencies [52].

### COVID and connections between equity, human rights, and democracy

An equity, human rights, and democracy lens clarifies how the COVID-19 pandemic amplified and exposed weaknesses in political and economic systems across the globe and deeply determined illness and death from COVID-19 not only between but also within countries. Across the globe, COVID-19 has had a more devastating effect where political leadership has been poor and unwilling to consider scientific evidence or heed the advice of medical experts. This association reinforces the importance of giving priority to health equity and human rights in designing fairer COVID-19 response strategies and policies and of specifically developing tools to challenge inequitable policies that disproportionately and unjustifiably cause harm to already oppressed and marginalised populations. While democracy did not ‘vaccinate’ against the election of populist leaders like Trump and Bolsonaro, it could provide some degree of constraint on their power. To the extent that human rights protections are somewhat synonymous with democratic rule, they can offer important tools within democratic states for civil society to challenge irrational and/or discriminatory health policies.

COVID-19 has vividly illustrated the theoretical idea that all human rights are interdependent [54]. On the one hand, this interdependence has deeply underscored the importance of having human rights principles and standards guide more equitable domestic and global COVID-19 responses [51,54,55,56]. On the other hand, it has illuminated that the protection of human rights is itself intertwined and partially dependent on the existence of legal mechanisms, political buy-in, and a free and active civil society, hallmarks to some extent of democratic societies. In this way, COVID-19 has illustrated what Goodhart has called a ‘negative interdependence’ in which a crisis triggers a ‘chain reaction of rights violations and deprivations that disproportionately affect oppressed and vulnerable people’ [56]. Experiences during COVID-19 underscore the need to focus on health governance at both the global and domestic levels and to integrate strong commitments to human rights and equity therein. Yet, COVID-19 equally underscores that commitments to equity and the existence of strong human rights protections will not themselves prevent racial stratifications in the impact of a pandemic disease, even in ostensibly rights-protective democracies like Canada. The study and practice of human rights, equity, and democratic governance needs to dig far deeper into the social, legal, and political biases that continue to drive and deepen health inequities, including within democracies.

These inequities and rights violations were part of the prompt for ongoing negotiations for a prospective pandemic treaty, which is being drafted to assure more effective global governance and cooperation in future pandemics. It is notable that the zero draft of this treaty, which will be negotiated over the next few years, highlights equity and human rights as guiding principles and standards for the domestic and global governance of future health emergencies. It prioritises equity and human rights in its vision, objectives (article 3), guiding principles (articles 4, 1, 2, and 4), and in pandemic prevention, preparedness, response, and recovery of health systems (chapter III). The treaty’s aim is to achieve

a world where pandemics are effectively controlled to protect present and future generations from pandemics and their devastating consequences, and to advance the enjoyment of the highest attainable standard of health for all peoples, on the basis of equity, human rights and solidarity …[and] to achieve greater equity and effectiveness for pandemic prevention, preparedness and response through the fullest national and international cooperation [22].

These are promising signs of growing awareness of the imperative for greater attention to equity and human rights in the governance of public health emergencies. Yet the extent of contestation around such clauses suggests that it is unlikely that they will survive the political negotiations to come.

## Conclusion

COVID-19 has surfaced many of the political, legal, and social determinants of health inequities globally. The pandemic has illustrated how rights-violating, negligent, and inequitable political leadership can deeply determine health outcomes. It has equally shown how democratic norms and institutions, including human rights and equity, offer discourse, standards, and tools that can be effectively used to challenge inequitable leadership on health. More fundamentally, it has shown how great the need is for approaches to public health emergencies rooted in human rights, equity, and good governance. The pandemic treaty in negotiation offers some hope that we will have stronger and more effective global structures in place to govern the next global pandemic. In their absence, there is little to suggest that in a future health emergency, the world will do any better.
